# Remodeler Catalyzed Nucleosome Repositioning: Influence of Structure and Stability

**DOI:** 10.3390/ijms22010076

**Published:** 2020-12-23

**Authors:** Aaron Morgan, Sarah LeGresley, Christopher Fischer

**Affiliations:** Department of Physics and Astronomy, University of Kansas, Lawrence, KS 66045, USA; am.morgan@ku.edu (A.M.); slegres@ku.edu (S.L.)

**Keywords:** chromatin remodelers, nucleosome repositioning, molecular machines, enzyme mechanism

## Abstract

The packaging of the eukaryotic genome into chromatin regulates the storage of genetic information, including the access of the cell’s DNA metabolism machinery. Indeed, since the processes of DNA replication, translation, and repair require access to the underlying DNA, several mechanisms, both active and passive, have evolved by which chromatin structure can be regulated and modified. One mechanism relies upon the function of chromatin remodeling enzymes which couple the free energy obtained from the binding and hydrolysis of ATP to the mechanical work of repositioning and rearranging nucleosomes. Here, we review recent work on the nucleosome mobilization activity of this essential family of molecular machines.

## 1. Introduction

**Figure 1:**
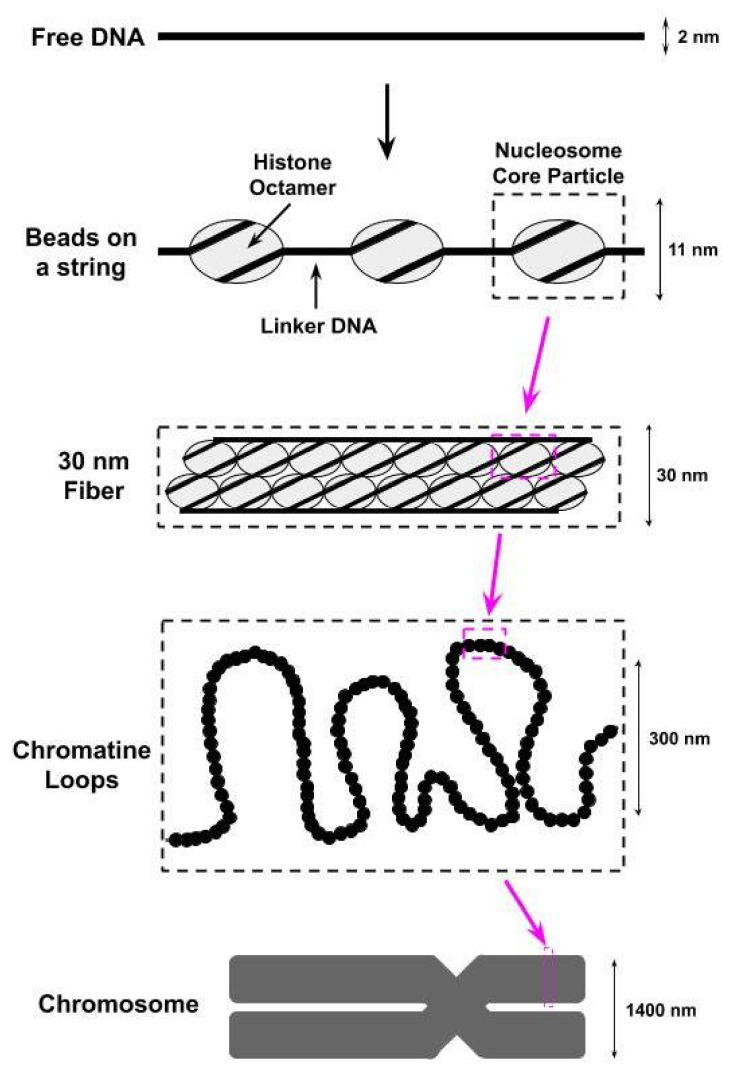
The first level of compacting the genome into chromatin involves wrapping the DNA around octamers of histone proteins to form nucleosomes. We identify in this figure a nucleosome core particle, which consists of an octamer of histone proteins wrapped by ≈147 basepairs of DNA. These nucleosome core particles are further wrapped together to form a structure known as a 30 nm fiber due to its physical dimensions. Supercoiling of the 30 nm fibers into chromatine loops then leads to the formation of a chromosome.

The compaction of the eukaryotic genome into chromatin begins with the wrapping (i.e., bending) of the DNA around octamers of the H2A, H2B, H3, and H4 histone proteins to form nucleosomes [1,2,3,4]; two copies of each of these histones are found within each octamer. The DNA wrapped within each nucleosome, referred to as nucleosomal DNA, contacts the histone octamer at 14 different sites, spaced approximately 10 basepairs apart, each of which contains several different types of noncovalent interactions (e.g., van der Waals interactions, hydrogen bonds) between the DNA and the histones [1,3]. The histone octamer together with the ≈147 basepairs of nucleosomal DNA wrapped around it is referred to as the nucleosome core particle (NCP). A single base pair of the nucleosomal DNA is centered on the nucleosome dyad and defines the pseudo-two-fold symmetry axis of the NCP [5]. DNA locations are designated by superhelical locations (SHL) representing superhelical turns from the dyad (SHL0) and ranging from SHL−7 to SHL7 [5,6]. As shown in Figure 1,the next level of chromatin packing is the ordering of nucleosomes into a “beads on a string” structure in which individual nucleosomes are separated by short stretches of free DNA, flanking either side of the NCP, that link NCPs together [7]; this free DNA is most often referred to as linker DNA, but is sometimes called flanking DNA.

Since the packaging of DNA within nucleosomes requires that the DNA be wrapped around the histone octamer and that specific contacts be made between the DNA and the histones [1,2,3,4,8], it is not surprising that the thermodynamic stability of a nucleosome depends upon the sequence of the wrapped DNA [9,10,11,12,13]; however, DNA sequence has little affect on nucleosome structure [2]. For example, sequences of DNA that are more easily bent display a higher affinity for octamer binding [14] and nucleosomes reconstituted using such DNA sequences are also more stable [14]. The periodicity of particular dinucleotides in DNA sequences with high affinity for histone octamer binding further demonstrates the correlation between DNA sequence and nucleosome stability as the positions of these dinucleotides in the DNA increase the flexibility of the DNA, thereby facilitating the wrapping the DNA around the histone octamer [9,10,14]. To that point, it is worth noting that in several studies of nucleosome structure, nucleosomal DNA accessibility, and chromatin remodeling activity, the NCP substrates were reconstituted using a sequence of DNA, referred to as the Widom 601 sequence, that was designed to have high affinity for nucleosome binding [9].

The wrapping of nucleosomal DNA around the histone octamer restricts the ability of DNA binding proteins to access it [15,16,17]. Consequently, the wrapping of nucleosomal DNA must be dynamically controlled in order to regulate the accessibility of DNA repair, DNA replication, and gene expression machinery [18,19]. One mechanism of control involves the activity of molecular motors called chromatin remodelers, which reposition NCPs along DNA (i.e., move histone octamers relative to the DNA) using an ATP-dependent mechanism [20,21,22]. Chromatin remodelers have been shown to play a role in many biological processes, from regulation of gene expression to DNA damage response, and to catalyze many enzymatic reactions, including nucleosome assembly and disassembly [20,23,24,25,26,27]. Our focus here is a summary of recent studies of the nucleosome repositioning activity of chromatin remodelers only. Our aim in this manuscript is to highlight common elements of chromatin remodeler structures and repositioning mechanisms such that these lead to generalized conclusions about this activity. We also discuss how the perturbations of the network of interactions between the chromatin remodeler, the nucleosomal DNA, and the histone octamer modulate this activity.

## 2. Structure

**Figure 2:**
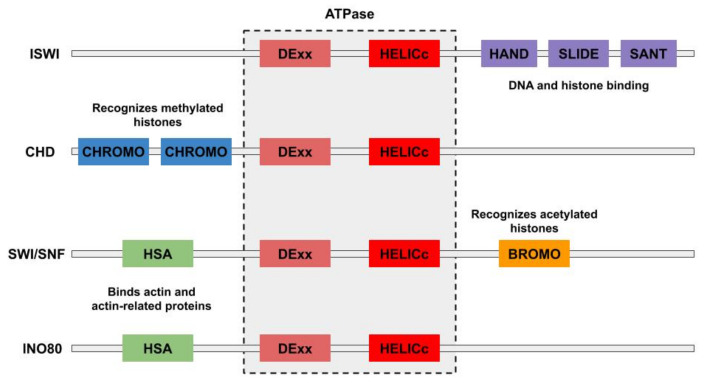
Pictorial representation of the domains conserved within each subfamily of chromatin remodelers. The DExx and HELICc domains are common to all chromatin remodelers and are found within the catalytic ATPase subunit. The HSA domain, responsible for binding actin and actin-related proteins, is common to both SWI/SNF and INO80 chromatin remodelers, but only SWI/SNF chromatin remodelers possess a BROMO domain, which is involved in recognizing acetylated histone proteins. The HAND, SLIDE, and SANT domains are unique to ISWI chromatin remodelers and play roles in DNA and histone binding. Only CHD remodelers possess CHROMO domains that are involved in recognizing methylated histones.

Based upon their highly conserved ATPase domain, chromatin remodelers are classified as part of the large Snf2 family of proteins, which in turn is part of the helicase superfamily SF2 [23,28]. This ATPase consists of two tandem RecA-like folds (DExx and HELIC) that allow for translocation along the minor groove of DNA [29]. Chromatin remodelers are further categorized into four subfamilies—ISWI (imitation switch), SWI/SNF (switch/sucrose-non-fermenting), CHD (chromodomain-helicase-DNA binding), and INO80 (inositol requiring 80)—based on additional domains that confer specific functional properties [20,23,24]; a summary of these domains and their functions is presented in Table 1 and Figure 2. SWI/SNF chromatin remodelers share an HSA (helicase SANT) domain and a bromodomain, which is involved in binding actin and actin related proteins, and recognizing acetylated histones [25,30]; INO80 chromatin remodelers also contain an HSA domain, but lack bromodomains [20,25]. SWI/SNF and INO80 chromatin remodeling complexes have also been shown to contain actin and/or actin-related proteins (Arps) that may play a role in regulating the assembly of the complex and its interaction with chromatin [31,32]. ISWI chromain remodelers contain the HAND, SANT, and SLIDE domains which play roles in DNA and nucleosome binding [33,34]. As suggested by its name, CHD chromatin remodelers contain chromodomains that recognize methylated histones [20,35]. Furthermore, each of these subfamilies consist of multiple ATPases, several of which have been shown to possess distinct chromatin remodeling activity [20,36]. The number of individual chromatin-remodelers is further increased by counting the numerous large multiprotein complexes that share in common one of these ATPases [20]. Indeed, *Saccharomyces cerevisiae* alone contains two SWI/SNF ATPases, Swi2/Snf2 and Sth1, that serve as the molecular motors for the ySWI/SNF and RSC chromatin remodeling complexes, respectively [37]; two different ISWI ATPases, Isw1 and Isw2, which are found in four different chromatin remodeling complexes [38]; one CHD family chromatin remodeler, Chd1 [39]; and one INO80 family chromatin remodeler [40,41]. A similarly large array of chromatin remodelers has been found in other species [25]. Please note that we use the generic name *chromatin remodeler* in this report whether we are referring to a monomeric enzyme or a multiprotein complex; the reader should always be aware, however, that most chromatin remodelers function as complexes.

The regulation of the nucleosome binding and repositioning activity of chromatin remodelers has long been a topic of considerable interest [30,34,38,42,43,44,45,46,47]. The results of a recent cryo-EM study showed that the ATPase domain of the *Chaetomium thermophilum* ISWI chromatin remodeler interacted with the NCP at the SHL2 location, causing a bulge in the nucleosomal DNA there [48]. This bulge of DNA may be a common element of ISWI interactions with NCPs as it is also observed in the ADP bound state of the complex of *Saccharomyces cerevisiae* Isw1 with an NCP [49]; indeed, as discussed below, it is possible that the formation of this bulge of DNA is the first step in the process of DNA translocation that must accompany NCP repositioning. The results from both of these studies further suggest that rearrangements of the AutoN and NegC regulatory domains within the catalytic core of ISWI accompany the binding of the enzyme to the NCP [48,49]. These structural rearrangements may be necessary to facilitate the conformational changes within ISWI that are required for binding the NCP [48,49]. The similarity between the structures of apo-Isw1 interacting with an NCP and ADP-bound Isw1 interacting with an NCP is consistent with previous reports that nucleotide binding by *Xenopus laevis* ISWI did not modulate the affinity of that remodeler to bind an NCP [50], and that the structure of the histone octamer was unaffected by the binding of *Chaetomium thermophilum* ISWI [48]. When taken together, these data suggest that ISWI forms a DNA bulge by effectively competing with the histone for DNA biding rather than primarily through distortions of the histones themselves. Additional subunits within ISWI subfamily chromatin remodelers are also known to play a role in nucleosome binding. For example, the HAND, SANT, and SLIDE domains have been shown to bind DNA flanking the NCP [33,34]. Since mutations in the SLIDE domain affect that ATPase and nucleosome repositioning activities of *Saccharomyces cerevisiae* Isw2, a mechanistic role for this domain beyond that of nucleosome binding is likely [51].

The *Saccharomyces cerevisiae* Snf2 remodeler has also recently been shown to interact with the NCP at either the SHL2 or SHL6 location [52,53,54]. When bound at SHL2, Snf2 generates a bulge of DNA at that location [52,53], similar to what is observed with ISWI binding to SHL2 [48,49]; as with ISWI, it is the ATPase domain of Snf2 that contacts the DNA at this location. The formation of this DNA bulge is allosterically regulated through nucleotide binding by Snf2; the bulge is present when ADP-bound SNf2 or apo-SNF2 bind the nucleosome, but absent when ATP-bound Snf2 binds the nucleosome [53]. This bulge is associated with distortion throughout the nucleosomal DNA that is consistent with translocation of the DNA with respect to the histone octamer. Since the formation and resolution of the DNA bulge is associated with nucleotide binding by Snf2, structural changes in the Snf-nucleosome complex associated nucleotide binding by Snf2 could modulate a process of DNA translocation by the enzyme [53]. Furthermore, the presence of a secondary DNA binding surface in Snf2 allows for additional interactions between the remodeler and the DNA, which can help stabilize nucleosome binding and may play a role in DNA translocation [52]. Indeed, the importance of multiple DNA binding sites to processive DNA translocation by genetically related helicases has been well documented [55,56]. Although the physical significance of Snf2 binding at the SHL6 location has not yet been determined, it is worth noting that studies of NCP binding by other remodeling enzymes have indicated that multiple remodelers can interact with the same NCP [50,57,58,59].

A recent cryo-EM structure of the SWI/SNF chromatin remodeler RSC bound to a nucleosome also shows an interaction between the remodeler’s ATPase domain with the nucleosomal DNA at the SHL2 location with additional contacts between the remodeler and the nucleosomal DNA occurring at the SHL−6 and SHL−7 locations [60]. Furthermore, interactions between the ATPase domain and the SHL2 location have also been observed for chromatin remodelers from the CHD subfamily [61,62] as well as supplementary interactions at SHL−6, SHL−7, or SHL7 [61,62]. In contrast, the ATPase domain of INO80 has been shown to interact with the nucleosomal DNA at the SHL−7 location with the remodeler making additional nucleosomal contacts as SHL2, SHL−2, SHL3, or SHL−3 [54,63]. This frequently observed binding of chromatin remodeler’s ATPase domains to the SHL2 location may result from the fact that the nucleosomal DNA has high plasticity at that location [1]; the flexibility of the DNA at that location may make it easier for the remodeler to compete with the histones for DNA binding there. Similarly, the nearly universal occurrence of a second location for remodeler binding to the NCP, especially one close to the entry site of the NCP (e.g., at SHL6 or SHL7) may suggest a mechanism for DNA binding and translocation associated with nucleosome repositioning that is common to members of multiple subfamilies of chromatin remodelers. It is unfortunate, however, that uncertainty about the stoichiometry of chromatin remodeler binding to nucleosome substrates in several kinetic studies of NCP mobilization injects ambiguity in the interpretation of the roles of different structural elements of these enzymes in the mechanisms of nucleosome binding and repositioning, as well as the process of nucleosome repositioning itself.

## 3. Mechanisms of Nucleosome Repositioning

Nucleosome repositioning is an important part of the epigenetic regulation of gene expression. Chromatin remodelers modify nucleosome spacing through the sliding of NCPs relative to the DNA or by removing/exchanging histones from the NCP [64]. The former process relies upon the ability of the chromatin remodeler to translocate the nucleosomal DNA. Indeed, chromatin remodelers share the ATP-dependent DNA translocation activity of other members of the helicase superfamily SF2 [23,28,65,66,67,68,69,70]. Chromatin remodelers lack the helicase activity [71] of other SF2 enzymes, however, since their ATPase domain lacks the wedge domain necessary for DNA strand separation [72].

The isolated ATPase subunit of the ISWI chromatin remodeler family has been shown to be an ATP-dependent DNA translocase as well as a functional chromatin remodeler [66,70,73]. Single-stranded DNA translocation by the ISWI ATPase has a 3′ to 5′ directional bias and low processivity [66,70]. This low processivity may result from an allosteric regulation of DNA binding by nucleotide binding as the binding of ATP reduces the DNA binding affinity of ISWI [50]. In contrast, NCP binding by ISWI shows much higher affinity and is not allosterically regulated by nucleotide binding [50,73]. These results thus provide further evidence that the HAND, SANT, and SLIDE domains share additional histone recognition responsibilities beyond their reported role in DNA binding [28]. It is also interesting that, although ISWI is a poorly processive DNA translocase, it is nevertheless remarkably efficient, moving an average of 14 nucleotides per ATP molecule hydrolyzed [70]. It should be noted, however, that non-uniform motion may inflate the estimate of this distance [70]. Regardless, DNA translocation by ISWI is more efficient than the 1–4 nucleotides translocated per ATP molecule hydrolyzed that has been reported for genetically related helicases [74,75,76].

The coupling of ATP hydrolysis to the DNA translocation activity of a minimal construct of the essential SWI/SNF-family RSC chromatin remodeler, consisting of three of the fifteen subunits of the full length RSC [77,78], has also been characterized [69]. This minimal construct, denoted as RSCt, consists of the ARP7 and ARP9 subunits together with a truncated version of the Sth1 subunit, which contains the DNA-binding ATPase and translocation motor [78,79]. Similar to what has been observed with ISWI, RSCt displays a 3′ to 5′ directional bias for DNA translocation [65,67] and low translocation processivity [69]. In contrast to what was observed for ISWI, however, translocation by RSCt is not as energy efficient as DNA translocation by ISWI or genetically related helicases [74,75,76], requiring the hydrolysis of three ATP molecules for each nucleotide translocated [69]. RSCt was also shown to undergo an initiation process following its binding to DNA before becoming competent for DNA translocation and to be capable of exerting enough force during DNA translocation to disrupt a biotin–streptavidin linkage [69].

It is also interesting to note that the macroscopic rates of DNA translocation by ISWI and RSCt are similar to the macroscpoic rate of DNA translocation by the NS3h helicase from hepatitis C virus [75], but much slower than the macroscopic rates of DNA translocation by the UvrD [80], Rep [81], and T7 [74] helicases. This may suggest an underlying difference between helicase superfamilies with SFII helicases (NS3h, RSC, and ISWI) translocating along DNA much more slowly than SFI (UvrD and Rep) or DNAB-like (T7) helicases. Of course, while these results are interesting from the standpoint of making general comparisons, it is difficult to draw distinctive conclusions from them as these studies of these enzymes were performed under different conditions.

**Figure 3:**
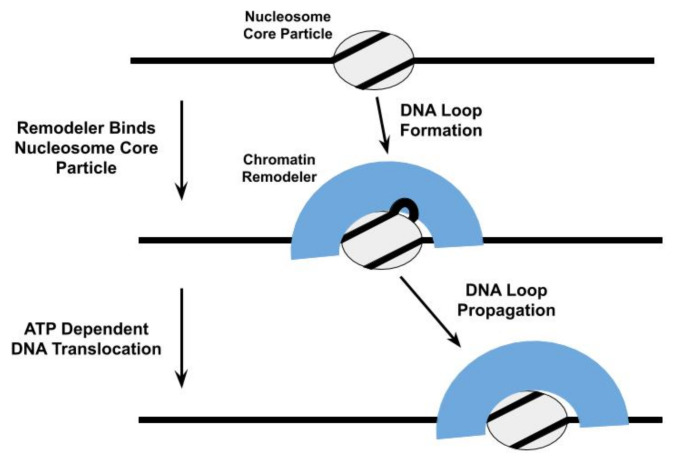
One model proposed for the repositioning of the nucleosome core particle involves the formation and propagation of DNA loops [49,53,65,67,87,88]. The propagation of these DNA loops relies upon the ability of the chromatin remodeler to translocate the nucleosomal DNA relative to the histone octamer.

DNA translocation by remodelers does play a central role in many models of their nucleosome repositioning activity [28,49,53,65,73,82,83,84,85]. For example, the ATPase subunit of the *Saccharomyces cerevisiae* SWI/SNF and RSC chromatin remodelers are proposed to bind to a specific site within the NCP and subsequently engage in directional DNA translocation, pulling in DNA from one side of the NCP and pumping it out from the other side, to shift the histone octamer relative to the DNA [86,87]; this DNA translocation proceeds through the formation and propagation of DNA loops [65,67,87,88], twist defects in the DNA [89,90], or some combination of both (Figure 3). Thus, the SWI/SNF complex initiates nucleosome repositioning by competing with the histone octamer for DNA binding. The proposal that DNA loops are an intermediate in the nucleosome repositioning mechanism of SWI/SNF is further supported by observations that *Saccharomyces cerevisiae* RSC forms DNA loops during its translocation along free DNA [69,77,91] and with the formation of DNA loops in Snf-nucleosome complexes being allosterically regulated through nucleotide binding by Snf2 [53].

DNA translocation plays a similar role in models of nucleosome repositioning by ISWI. Based upon single-molecule experiments, a “power-stroke” model has been proposed for the DNA translocation associated with nucleosome repositioning by *Saccharomyces cerevisiae* Isw1 and Isw2 [85]. According to this model, the chromatin remodelers slide the DNA relative to the histone octamer through a coordinated mechanism controlling DNA entering or exiting the NCP [85]. The chromatin remodeler is bound to the linking DNA on either side of the NCP and initiates the repositioning reaction by translocating the nucleosomal DNA one basepair at a time toward one end of the NCP through an ATP-dependent mechanism, building up strain within the NCP between the DNA and the histones. After this strain becomes sufficiently strong, corresponding to seven basepairs of translocated nucleosomal DNA, a conformational change occurs between the linker-DNA-binding domain of the chromatin remodeler (possibly the SLIDE domain) and the ATPase domain, which pushes three basepairs of DNA into the NCP. This allows three basepairs of DNA to move outside the NCP from the other side of the NCP. This process then repeats in units of three basepairs translocated per translocation cycle. This model is consistent with the results of more recent ensemble experiments [92], theoretical analysis [93], and with the formation of DNA loops in Isw1-nucleosome complexes being allosterically regulated through nucleotide binding by *Saccharomyces cerevisiae* Isw1 [49]. In an alternative ‘ratchet’ model for nucleosome repositioning by ISWI, the ATPase domain of the ISWI complex is responsible for all mechanical processes associated with nucleosome repositioning [73]. Remodeling still originates from the seven base pairs of flanking DNA being pulled through the nucleosome by the ATPase domain which causes internal tensions that must be resolved through the breaking and reforming of connections between the histones and the nucleosomal DNA [73]. This model assumes that the HAND, SANT, and SLIDE domains are responsible for passive histone recognition and the removal of the NegC domain from the ATPase cleft [73,94,95]. In each of these two models for nucleosome repositioning by ISWI, multiple rounds of ATP hydrolysis are required to reposition the nucleosome. Although additional work is required to further discriminate between these two models, it is worth noting that the step-size observed for DNA translocation by isolated ATPase subunit of *Xenopus laevis* ISWI is similar to what is predicted for the initial step in the ‘ratchet’ model [70]. Lastly, the *Saccharomyces cerevisiae* INO80 chromatin remodeler is capable of moving an NCP from an end position to a more central position along DNA and may serve as a nucleosome spacing factor [96]. Although it has been proposed that NCP binding by INO80 depends upon the length of the DNA flanking the NCP [96], that assessment was made without a concomitant determination of the stoichiometry of INO80 binding to the NCP. Indeed, it is possible that the changes in the apparent $K_{d}$ of NCP binding by INO80 associated with increasing length of flanking DNA may be attributed to additional biding sites on the flanking DNA itself, as was observed for ISWI [50].

Although ATPase activity is required for remodeler function, several studies have shown that the movement of the histone octamer relative to the DNA is poorly coupled to ATP hydrolysis for *Drosophila* ACF [97], human Snf2H [98], and *Xenopus laevis* ISWI [92]. This poor coupling efficiency between ATPase activity and octamer movement could be a fundamental property of these motors (i.e., these motors are inherently inefficient at nucleosome repositioning) or it could result from significant ATP hydrolysis being associated with either futile repositioning or the initiation of repositioning. A large energetic cost for initiating the repositioning reaction seems unlikely as the bulges of DNA suggested to be the precursors of DNA translocation and nucleosome repositioning can be formed by chromatin remodelers binding to nucleosomes in the absence of ATP hydrolysis [48,49,53]. Since DNA translocation is fundamental to most models of nucleosome repositioning, DNA translocation has been proposed to be the energetically rate-limiting process during nucleosome repositioning [99]. However, since DNA translocation by chromatin remodelers is remarkably energy efficient [70], it seems unlikely that DNA translocation itself would be significantly less energy inefficient when coupled to nucleosome repositioning. However, since nucleosome repositioning requires the chromatin remodeler to break and reform contacts between the histones and the nucleosomal DNA in order for DNA translocation to occur, disrupting the interactions between the histones and the nucleosomal DNA does provide an energetic barrier to nucleosome repositioning. This barrier, if sufficiently large, could result in multiple rounds of unsuccessful attempts at nucleosome repositioning and the associated DNA translocation for each successful repositioning event [92,93]. This hypothesis could also explain the poor template commitment reported for human Snf2H [98] as well as the observation that *Xenopus laevis* ISWI repositions nucleosomes through a random walk mechanism [92]. Indeed, since most studies of chromatin remodeler function involve NCP substrates reconstituted using the Widom 601 DNA sequence [9], it is possible that experiments intended to assess the repositioning activity of chromatin remodelers, may instead report primarily on the affinity of histone:DNA interactions within the nucleosome rather than the activity of the remodeler. This proposition is further supported by the observation that the rate of nucleosome repositioning by *Xenopus laevis* ISWI depends upon the sequence of the nucleosomal DNA, with faster repositioning occurring with DNA sequences with lower affinity for histone binding [93]. Indeed, if the DNA translocation associated with nucleosome repositioning requires the chromatin remodeler to compete with the histones for binding the nucleosomal DNA [49,53,87], then it should be more difficult for chromatin remodelers to reposition nucleosomes reconstituted with DNA sequences with higher affinity for histone binding [93]. It is therefore not surprising that several studies have shown that the sequence of nucleosomal DNA influences the nucleosome repositioning activity of chromatin remodelers [92,100,101,102,103]. It is worth noting that the nucleosome repositioning activity of *Saccharomyces cerevisiae* INO80 has been reported to be independent of the sequence of nucleosomal DNA [104], but a lack of determination of the stoichiometry of INO80 binding to the NCP substrates used in these experiments creates some ambiguity in the interpretation of those results, especially for nucleosome substrates containing long lengths of DNA flanking the NCP [50,92]. While a dependence of nucleosome repositioning on the sequence of nucleosomal DNA may be helpful in providing an alternative tool for measuring DNA–histone interactions (i.e., an alternative probe of histone:DNA interaction free energy [10]), it clearly muddles what can be concluded about the intrinsic repositioning activity of the remodeler and how this activity is influenced and modulated [93].

## 4. Effects of Substrate Modification On NCP Binding and Mobilization

**Figure 4:**
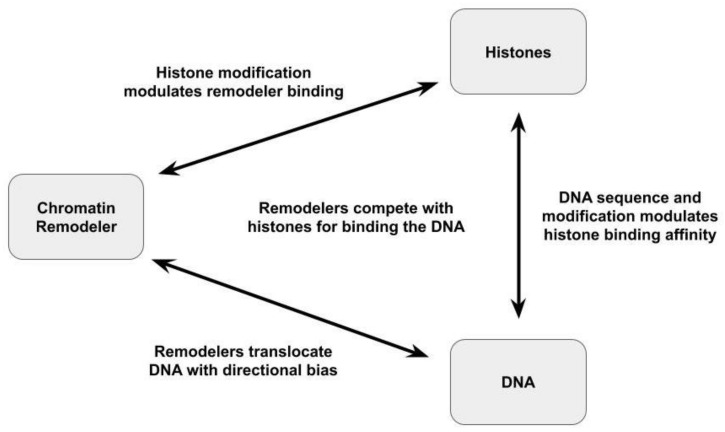
The network of interactions between the DNA, histones, and the chromatin remodelers that influence nucleosome repositioning.

As discussed above, nucleosome repositioning requires the chromatin remodeler to compete with the histones for DNA binding so that DNA translocation can occur. As shown in Figure 4, nucleosome repositioning can thus be modulated by modifications within the network of interactions between the chromatin remodeler, the histones, and the DNA. Indeed, regulating the affinity of NCP binding and NCP mobilization (e.g., by affecting DNA translocation) through histone and/or DNA alterations is the basis of the proposal for kinetic proof-reading by chromatin remodelers [105,106,107]. For example, decreasing the affinity of NCP binding (i.e., increase the rate of dissociation from the NCP) due to a loss of contacts with the H4 tail or parts of the flanking DNA decreases the overall rate of NCP movement for human ACF1 [106]. Similarly, the strong affinity of histone binding by the Widom 601 sequence has been shown to modulate the NCP repositioning activity of *Xenopus laevis* ISWI [50,92]. In that case, the energetic barrier associated with disrupting histone:DNA contacts often prevented successive rounds of NCP movement before ISWI dissociation.

The ability of post-translational modification of histone proteins to influence interactions between chromatin remodelers and their nucleosome substrates provides an epigenetic mechanism of regulating the activity of these enzymes. Indeed, acetylation and methylation of the H3 and H4 histones is known to affect interactions between chromatin remodelers and NCPs [30,35,38,108,109,110] and thus serves as an regulator of transcription [111,112,113,114]. It is interesting to note that although the removal of the N-terminal tails of the H2A, H2B, and H3 histones do not affect the nucleosome repositioning activity of the ISWI subfamily chromatin remodelers CHRAC and NURF, removal of the N-terminal tail of the H4 histone blocks nucleosome repositioning by these remodelers [109,115,116]. Similarly, removal of the N-terminal tail of the H4 histone reduces the nucleosome repositioning activity of other ISWI and CHD subfamily chromatin remodelers [73,117,118,119]. Since the N-terminal tail of the H4 histone is at the SHL2 location, which, as discussed above, is where the ATPase domain of many chromatin remodelers bind the NCP, it is not surprising that it is required for the binding of *Saccharomyces cerevisiae* ISW2 to SHL2 [117]. In contrast, deletion of the N-terminal tail of the H4 histone was shown to reduce the nucleosome repositioning activity of the SWI/SNF subfamily chromatin remodeler RSC and the CHD subfamily chromatin remodeler Chd1, without reducing the affinity of NCP binding by those enzymes [118]. While these latter results suggest a possible mechanistic role for this tail in addition to its role in regulating NCP binding affinity, it is important to note that binding affinity was assessed through the Michaelis–Menten parameter $K_{M}$ [118] rather than equilibrium measurements of NCP binding and the allosteric regulation of it by nucleotide binding. Lastly, the sequence of the auto-inhibitory region of ISWI, denoted as AutoN, has high similarity to the sequence of the N-terminal tail of histone H4 [94]. When ISWI binds to an NCP, the ATPase domain of ISWI interacts with the N-terminal tail of histone H4, rather than AutoN [48].

Modifications of the N-terminal tail of histone H3 have also been shown to affect NCP binding and mobilization. The SWI/SNF and ISWI family chromatin remodelers were shown to bind H3K9AcK14Ac-containing di-nucleosomes with significantly higher affinity than unmodified di-nucleosomes [120]. Since these complexes consist of multiple subunits, each of which with a separate affinity for binding modified and unmodified di-nucleosomes, it is important to recognize that the binding behavior of a given remodeler complex is a convolution or superposition of the binding behaviors of its subunits. Indeed, the PHF10 subunit of SWI/SNF appears to preferentially interact with H3K9AcK14Ac, displaying one of the highest affinities for this modification of all SWI/SNF family subunits, while seeming to be repelled by H3K4me3 [120]. In contrast, the SMARCA2 and SMARCA4 subunits of SWI/SNF engage in high affinity interactions with H3K4me3 modified di-nucleosomes [120]. The ISWI catalytic subunit SMARCA5 and accessory subunits BPTF, C17orf49, BAZIA, and BAZIB display relatively high affinity for both modified and unmodified di-nucleosomes, with SMARCA5 preferentially binding H3K9AcK14Ac-modified over H3K4me3-modified or unmodified di-nucleosomes [120]. It is worth noting, however, that although these accessory subunits all show high affinity for binding these modified substrates, they each exhibit distinct binding interactions with them [120]. Similarly, the binding behaviors of complexes such as NuRF, ACF, and WICH—each containing a different subset of SMARCA5 and the previously mentioned ISWI family accessory subunits—likely represent convolutions of the binding behaviors of their respective subunits [120]. Clearly, additional characterizations of the binding contributions of the constituent subunits of chromatin remodeling complexes can provide further insight into not only how these complexes are regulated by post-translational modifications, but also into their nucleosome binding and repositioning activities themselves. Such studies could also identify common elements of behavior and regulation. For example, is it the case that a complex containing SMARCA2/4 will always see an increased affinity for H3K4me3, while a complex containing PHF10 will always see a reduced affinity? If so, that what evolutionary pressures led to the incorporation of different subunits into different complexes?

Amino acids within the H2A and H2B histones create a negative charged region on the surface of the NCP [1]. This acidic patch has been shown to be a target for several nucleosome-interacting proteins and to play a role in the formation of the higher-order chromatin structures shown in Figure 1 [121,122,123]. Indeed, several chromatin remodelers have been shown to possess a region of positive charge, commonly referred to as a basic motif, that interacts directly with the NCP acidic path during NCP binding [8,124]. One example of this is the previous mentioned AutoN and NegC domains of the ISWI subfamily chromatin remodeler SNF2h, which have been shown to interact directly with the acidic patch of the NCP [125]. Mutations in either the AutoN or NegC domains reduce the requirements of the negative charges in the acidic patch of the NCP for optimal nucleosome repositioning [125]. Decreasing the negative charge within the acidic patch also decreases the rate of nucleosome repositioning by the *Saccharomyces cerevisiae* INO80 chromatin remodeler [125]. It is also worth noting that the chromatin substrates of the INO80 subfamily include the histone H2A variants H2AZ and H2AX, and the interaction between INO80 and phosphorylated H2AX within the nucleosome is mediated by INO80’s Nhp10 and Arp4 subunits [126]; the histone variants H2AZ and H2AX are implicated in numerous cellular processes through their roles in regulating transcription and DNA repair [127,128,129].

Different chromatin remodeler complexes, including complexes possessing differing ATPase domains and accessory proteins, establish unique nucleosome positioning patterns [100,130]. For example, the final NCP positions resulting from nucleosome repositioning by the human Snf2H remodeling complex depended upon whether the Tip5 domain was present in the complex [100,101]. Similarly, studies employing electrophoretic mobility assays to compare the repositioning activity of a range of chromatin remodelers—Brg1, Chd1, ISWI, Snf2H, Mi-2, ACF, INO80, and NURF—showed that each has its own preference for repositioning nucleosomes into a particular set of positions (i.e., nucleosome spacing). Because it appears that intermediate positions remain mostly identical to initial nucleosome positions it has been suggested that positioning patterns are the result of unique interpretations of DNA sequence/structure information, resulting in unique distributions of affinity for different nucleosome positions by chromatin remodelers with differing ATPase motor proteins and non-catalytic accessory proteins [100]. For example, *Xenopus laevis* ISWI was shown to move NCPs between positions corresponding to minimum energy states in the interactions between the histones and the DNA [92,93]. However, the panoply of interactions between the histones, the nucleosomal DNA, and the chromatin remodeler (Figure 4) has yet to be fully and independently characterized for any system. In the absence of such a characterization, it is not possible to conclude that the pattern of nucleosome positioning a particular chromatin remodeler displays is wholly a result of the effects of nucleosome position on the affinity of nucleosome biding by the remodeler and not—at least in part—a result of the remodeler’s unique capability for disrupting the binding of particular sets of nucleosomal DNA sequences to the histone. Indeed, the apparent poor coupling of NCP repositioning to ATP binding and hydrolysis for *Drosophila* ACF [97], human Snf2H [98], and *Xenopus laevis* ISWI [92] suggests that nucleosome repositioning patterns are likely influenced by more than a remodeler’s affinity for a set of substrates, consistent with kinetic proof-reading models [105,106,107]. Furthermore, the genome-wide distributions of *Drosophilia* NURD, (P)BAP, INO80, and ISWI chromatin remodelers indicate that each of these chromatin remodelers has a specific set of repositioning targets and generates distinct nucleosome distributions [131], likely resulting from variations in affinity of genomic DNA for binding the histone octamer and variations in the location concentration of the chromatin remodelers and their affinity for NCP binding, including its regulation by post-translational modifications.

The fact that the sequence of nucleosomal DNA influences the nucleosome repositioning activity of chromatin remodelers [92,93,100,101,102,103] provides another mechanism of regulating the activity of these enzymes. Of particular interest is the role of chromatin remodelers in DNA damage detection and repair. For example, the NuRD complex and, in particular, its subunit CHD4 have been shown to play a role in the repair of double-stranded DNA breaks [132,133]. Similarly, the INO80 chromatin remodeler is proposed to play a role in in homologous recombination and DNA replication by preferentially binding to four-way junctions or three-way junctions and evicting nucleosomes near double stranded breaks [126,134]. While histone modifications may be responsible for some of the regulation of chromatin remodeling activity as part of DNA damage detection and repair [135], it has also been suggested recently that DNA damage induced changes in the free-energies of interactions among the DNA, the histones, and the chromatin remodeler may favor chromatin remodelers repositioning nucleosomes to locations where the DNA damage can be more easily detected and repaired [93,136]. Thus, the thermodynamics associated with the packaging of DNA into nucleosomes might allow DNA damage to facilitate its own detection, which could provide eukaryotes with a significant evolutionary advantage.

## 5. Outlook

The nucleosome repositioning activity of chromatin remodelers is an emergent property of variations within the network of interactions among the remodeler, the nucleosomal DNA, and the histones, with the interactions of the chromatin remodelers further allosterically regulated by ATP binding and hydrolysis. Post-translational modifications of the histones and/or the chromatin remodelers include specific targeting of subunits or accessory proteins within the chromatin remodeler, and variations in DNA sequence provide a further mechanism of control and regulation. In light of this, future studies of the nucleosome repositioning activity of chromatin remodelers would benefit from a systematic and holistic characterization of the interactions in this network. This should include coordinated studies of nucleosome binding and repositioning so that the fraction and identification of bound species in the repositioning reaction is known [50,92]. These experiments should also be conducted using a range of sequences for the nucleosomal DNA and a variety of histone post-translational modifications, coupled with independent determinations of the stability of each nucleosome substrate, so that changes in the rate of nucleosome repositioning can be correlated with changes in the energetics of the remodeler-NCP interaction or nucleosome stability [93]. In addition to being the most effective pathway to characterizing correctly the NCP binding and mobilization activity of chromatin remodelers, such an approach would also be applicable for future studies of how chromatin remodelers interact with other elements of the cell’s DNA transcription, repair, and replication machinery to regulate these essential biological functions [105].

## Figures and Tables

**Table 1:** Subfamilies of chromatin remodelers.

Subfamily	Principle Activity	Additional Domains	Domain Function
ISWI	Nucleosome assembly and spacing Transcription regulation	HAND, SANT, and SLIDE	DNA and nucleosome binding
SWI/SNF	Transcription regulation	HSA BROMO	Binds nuclear actin-related proteins Recognizes acetylated histones
CHD	Interacts with promoter DNA sequences Transcription regulation	CHROMO	Recognizes methylated histones
INO80	Inositol-responsive gene expression Deposition of histone variant H2AZ	HSA	Binds actin and actin-related proteins
